# New Pandemic: Obesity and Associated Nephropathy

**DOI:** 10.3389/fmed.2021.673556

**Published:** 2021-06-29

**Authors:** Isha Sharma, Yingjun Liao, Xiaoping Zheng, Yashpal S. Kanwar

**Affiliations:** ^1^Departments of Pathology and Medicine, Northwestern University, Chicago, IL, United States; ^2^Department of Nephrology, Second Xiangya Hospital, Central South University, Changsha, China; ^3^Department of Urology, Third Xiangya Hospital, Central South University, Changsha, China

**Keywords:** obesity, kidney, hyperlipidemia, inflammation, oxidant stress, fibrosis

## Abstract

Incidence of obesity related renal disorders have increased 10-folds in recent years. One of the consequences of obesity is an increased glomerular filtration rate (GFR) that leads to the enlargement of the renal glomerulus, i.e., glomerulomegaly. This heightened hyper-filtration in the setting of type 2 diabetes irreparably damages the kidney and leads to progression of end stage renal disease (ESRD). The patients suffering from type 2 diabetes have progressive proteinuria, and eventually one third of them develop chronic kidney disease (CKD) and ESRD. For ameliorating the progression of CKD, inhibitors of renin angiotensin aldosterone system (RAAS) seemed to be effective, but on a short-term basis only. Long term and stable treatment strategies like weight loss via restricted or hypo-caloric diet or bariatric surgery have yielded better promising results in terms of amelioration of proteinuria and maintenance of normal GFR. Body mass index (BMI) is considered as a traditional marker for the onset of obesity, but apparently, it is not a reliable indicator, and thus there is a need for more precise evaluation of regional fat distribution and amount of muscle mass. With respect to the pathogenesis, recent investigations have suggested perturbation in fatty acid and cholesterol metabolism as the critical mediators in ectopic renal lipid accumulation associated with inflammation, increased generation of ROS, RAAS activation and consequential tubulo-interstitial injury. This review summarizes the renewed approaches for the obesity assessment and evaluation of the pathogenesis of CKD, altered renal hemodynamics and potential therapeutic targets.

## Introduction

“Diabesity” is a new term, often used globally, to reflect the status of coexisting diabetes and obesity, especially those in the aging population. It has emerged as the major cause of chronic kidney disease (CKD) (1–7). World health organization (WHO) data suggest that since 1975 worldwide obesity (BMI ≥30 kg/m^2^) has tripled. According to 2016 statistics over 1.9 billion adults, i.e., 18 years of age and older, were obese, and nearly 39% of them were overweight. Among the obese population about 13% were men and 15% and were women, respectively, across diverse ethnicities. Wide ethnic disparities were found for the prevalence of obesity: Whites (22.0%), Latinos (33.6%), African Americans (36.1%), and Asians (9.8%) (8). In recent years, childhood obesity has also become more prevalent, and nearly 38 million children under the age of five have been found to be obese worldwide. Moreover, over 340 million children and adolescents, between the ages of 5–19, were regarded as obese worldwide. In view of the increasing incidence of obesity it is estimated that by the year 2030 more than 50% of the USA population will be obese (9). Sadly enough, this epidemic of obesity and its associated complications would cause a substantial socioeconomic burden to various countries. In addition, increase in the incidence of obesity has led to some of the associated systemic chronic disorders; such as nephropathy that is characterized by proteinuria, subclinical inflammation, progressive fibrosis and ultimately resulting in the decline of renal functions. In general, obese individuals are more adversely affected globally than those with normal- or under-weight. To gage characteristics of obesity, elevated BMI is used as one of the major parameters (10), however, more ancillary stringent methods for the assessment and management of obesity are needed. This review focusses on recent developments in the assessment of obesity, its pathogenesis and complications, including perturbed fatty acid metabolism, altered renal hemodynamics and development of CKD. Lastly, potential therapeutic targets, which can be used for the amelioration of obesity and its associated renal complications are discussed.

## Obesity Induced Diabetic Nephropathy

### Obesity: Definition and Epidemiologic Analysis

According WHO guidelines individuals with BMI between 20 and 25 kg/m^2^ are considered normal, and having BMI between 25 and 30 kg/m^2^ as overweight, and with >30 kg/m^2^ are regarded as obese. Based on the BMI, obesity itself has been classified into three grades: grade 1, 2, 3 with BMI being 30–34.9 kg/m^2^, 35–39.9 kg/m^2^, and ≥40 kg/m^2^, respectively (11, 12). A study was conducted at the Columbia University correlating high BMI and CKD over a span of three decades, and it pointed toward a gradually increasing incidence of the latter, i.e., CKD, from 0.2% in 1986–1990 to 2% in 1996–2000, which further increased to 2.7% in 2001–2015 (12). The data also indicated that 56% of the patients had proteinuria, while 44% had both proteinuria and a decline in renal functions (11, 12). Although this correlation may be applicable to Western population, it should be noted that among the Asians even people with lower BMI have the potential risk of developing CKD (12). Here, another enigmatic issue is that although BMI is easy to calculate, it is not an ideal predictor of CKD, and it may not accurately reflect the spatial distribution of fat mass with respect to obesity (13, 14). Moreover, at times, it is hard to distinguish between BMI of an individual with heavy musculature and one having ample subcutaneous fat; ironically both may have the same BMI. Certainly, the one with excessive amount of visceral fat may have the higher risk for the development of CKD (15). As an alternative to traditional BMI measurement, waist circumference (WC) and waist hip ratio (WHR) estimates would be more accurate reflective of the extent of visceral fat depots. In this regard, it is known that the incidence of obesity associated diabetes or CKD the incidence increases with a WC >102 cm and WHR of 0.9 for men, and >88 cm and 0.8 for women (15). Also, WHR and skin fold thickness are better predicators of CKD in obesity compared to BMI (16).

### Overview of Obesity and Associated CKD

**Figure 1:**
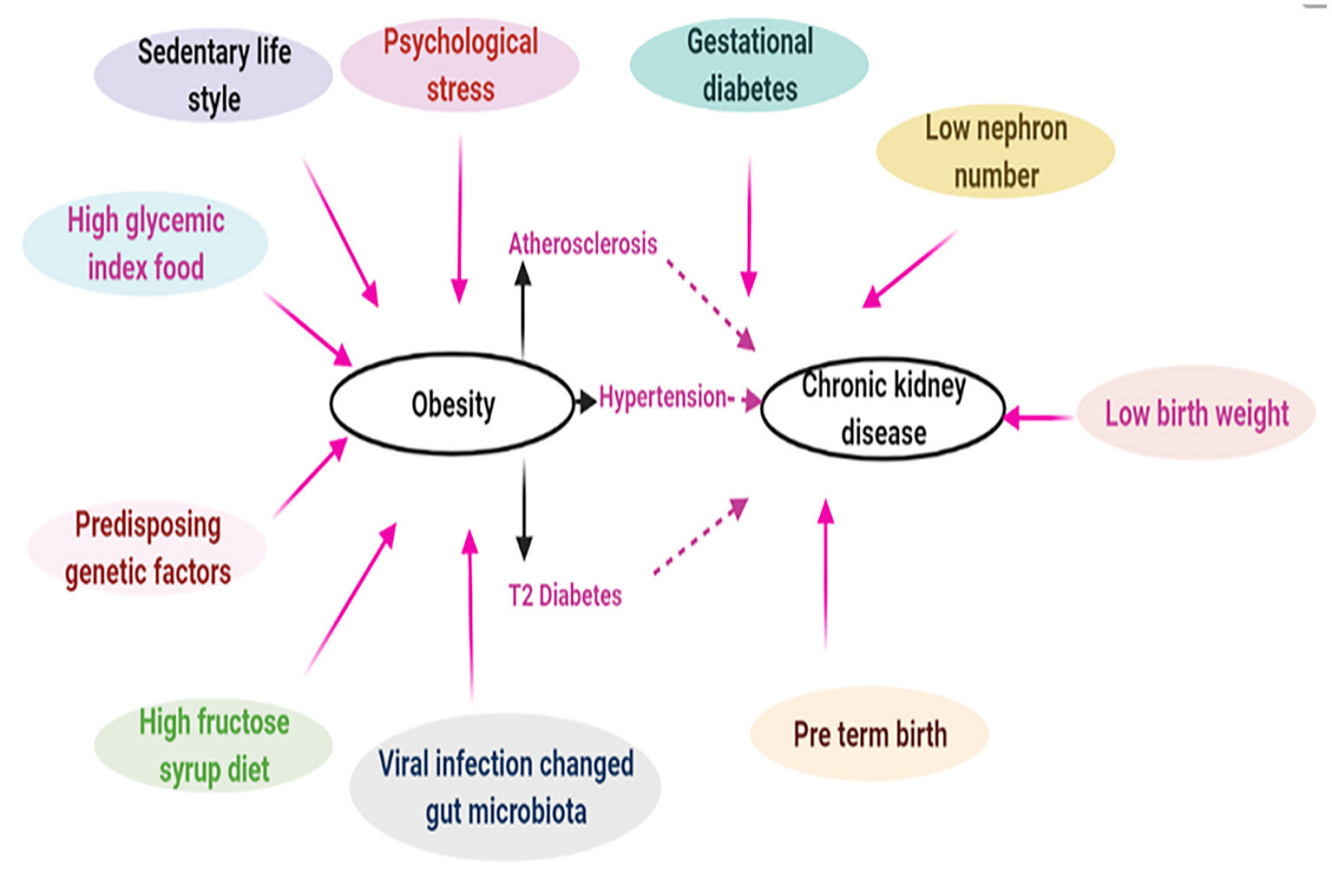
Complex intricate relationship between obesity and chronic kidney disease. Pathophysiology of obesity is very complex which may include genetic (epigenetic) and environmental factors. Obesity driven hypertension, atherosclerosis, and T2 diabetes may lead to chronic kidney disease. In addition, other ancillary factors like gestational diabetes, low nephron number can also contribute to CKD [Adapted from Stenvinkel et al. (2)].

In recent years, obesity has become a considerable health problem that has affected people of all races and ethnicities. Even if society becomes aware of this huge socioeconomic problem, it still may take decades to revert the increasing incidence of obesity to the prevailing levels of 30–40 years ago. There was a misconception that the rapid increase in obesity is observed only in well-developed nations, such as Europe and USA (17). On the contrary, countries with low and middle-income strata are experiencing a similar dilemma. For instance, recent data indicate that the prevalence of obesity and CKD have markedly increased in countries like China (18), India (19), and Brazil (20). Customarily, individuals with poor knowledge of nutrition and with substandard living conditions are at a greater risk for developing obesity and its related complications. Clinical data suggest that obesity and obesity associated glomerulopathy (ORG) are potential risk factors for the development and progression of CKD. It is worth mentioning here that the data of retrospective clinical study on renal biopsies of 620 diabetic patients suggested a high proportion of them, i.e., 33%, had diabetic nephropathy, and in addition 27% had diabetic nephropathy with superimposed “non-diabetic renal disease” (12, 21). Before discussing the pathogenesis of CKD comprehensively, a general overview of the pathophysiology of obesity may be worthwhile considering. It is a complex process with a multitude of inter-related facets, including genetic predisposition, individual dietary habits and physical inactivity, and environmental and other ancillary factors (Figure 1). First, until recently, 150 gene loci have been linked to the development of obesity and type 2 diabetes (22). Fat-mass and obesity-associated gene (FTO) polymorphism, localized to chromosome 16, has been described to be associated with ~7% phenotypic variability in BMI (23). Although much advancements have been made in identifying the epigenetic markers of obesity, there is still a lack of information regarding tissue specific epigenetic markers of obesity (24). Here, a thorough understanding of energy balance/imbalance is needed since it is one of the key factors responsible for obesity. Considerable intake of sucrose and fructose over a long period certainly increases the risk of developing metabolic syndrome with consequential CKD (25, 26). Ironically, the gratuitous marketing strategy by the food industry of enriching certain products with high fructose (27), such as corn syrup, with high glycemic index is not very helpful; rather it exacerbates the epidemic of obesity, thereby increasing the incidence of a number of metabolic associated disorders, e.g., type 2 diabetes, hypertension, gout and CKD (26, 28). Moreover, a sedentary lifestyle with physical inactivity further contribute to the development of obesity and its related disorders (29). Other factors, which could serve as conduits for the onset of obesity and subsequent CKD may include, neurocognitive factors (30), psychological stress (31), genetic mutations (32), altered gut microbiota (33) and adenoviral infections, e.g., adeno virus-36 (AD-36) (34). Ancillary background factor(s) responsible for the development of CKD may include reduction in the nephron number (35). In this regard, children with prematurity and intrauterine growth retardation of the fetus are also considered as risk factors for the onset of CKD following development of hypertension and T2DM in later life. Indeed, epidemiological studies support the data that a combination of low birth weight, malnourished childhood, and adolescent obesity would augment the prevalence of CKD (36).

### Hyperlipidemia Induced Renal Injury

The extent of ectopic lipid accumulation positively correlates very well with insulin resistance compared to any other predicators of obesity onset, i.e., BMI, WC, or WHR (37). Such lipid accumulation expectedly adverse the pathobiology of renal cells. For instance, with the excessive lipid accumulation the renal glomerular mesangial cells, and at times the endothelial cells, undergo foamy transformation via various receptor-mediated mechanisms with a multitude of adverse downstream events (38). In this regard, *in vitro* studies have shown progressive loss of contractile functions of mesangial cells with lipid accumulation. In the event where there is a concomitant subclinical inflammation, such as in diabetes, the lipid accumulation would accentuate, and which may lead to LDL-receptor dysregulation and disruption in feedback regulation and foamy transformation of intrinsic renal glomerular cells (podocytes, endothelial, and mesangial cells) as well as accumulation of triglycerides (39). The latter may accentuate dysfunctions of the glomerular cells, altered hemodynamics, increased nitric oxide (NO) generation, augmented inflammation and subsequent albuminuria (39, 40).

**Figure 2:**
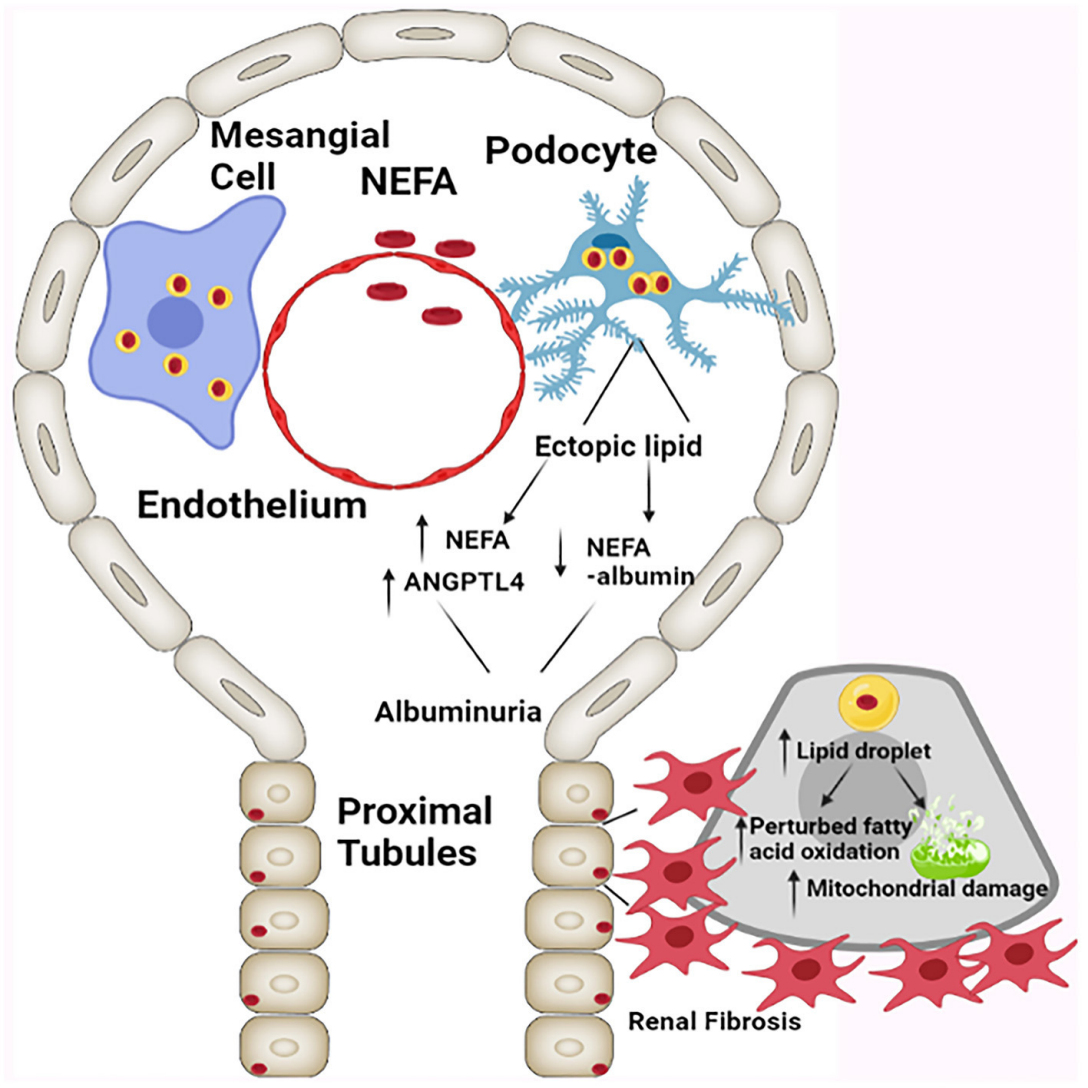
Schematic presentation depicting the effect of ectopic cellular lipid accumulation resulting in the compromise of glomerular and tubular integrity, and causing albuminuria, altered mitochondrial dynamics, and tubulo-interstitial fibrosis. NEFA: Non-esterified fatty acids, ANGPTL4: Angiopoietin-related protein 4 [Adapted from D'Agati et al. (12)].

The hydrolysis of triglycerides leads to formation of glycerol and non-esterified free fatty acids (NEFA). Normally, a majority of the NEFA/FFA are albumin bound and induce macropinocytosis in podocytes, which would be reflective of their functional integrity (40, 41). Conversely, free fatty acids (FFA) are likely to induce renal cellular damage to the podocytes with consequential albuminuria. Incidentally, ratio of free serum NEFA to albumin bound NEFA increases in parallel with angiopoietin-related protein 4 (ANGPTL4), the latter inhibits lipoprotein lipase leading to further triglyceridemia and inducing a proteinuric response (40, 41). Onset of hyperlipidemia and albuminuria in obesity is a self-perpetuating loop (Figure 2), as hypothesized by Moorhead and others, which state that lipid accumulation and infliction of renal injury are mutually interdependent processes enhancing the activity of each other (42, 43). Increased fat deposition not only leads to a compromise in the functionality of mesangial cell and podocytes, but it also initiates a series of downstream cellular events with activation of TGF-β/Smad3 signaling pathway, which ultimately lead to renal fibrosis. The renal fibrosis encompasses both glomerulosclerosis and tubulo-interstitial fibrosis. With respect to the latter, proximal tubules, heavily consume NEFA that are needed for meeting the demands of energy-dependent tubular transport processes. However, in states of obesity there may be availability of excessive amounts of NEFA which are likely to accumulate in the cytosolic lipid droplets as triglycerides (42, 44). This amassing would eventually perturb other cellular processes, including fatty acid oxidation. Here, although it has been reported that obesity associated renal damage is linked to perturbed fatty acid metabolism (45), the question arises as to how these cellular perturbations eventually cause tubulo-interstitial fibrosis. Supporting the above-mentioned notion, deep sequencing analysis in patients with CKD suggested impairment in fatty acid oxidation and activation of pathways related to inflammatory processes (45). The proximal renal tubular epithelia are mainly dependent on fatty acid oxidation (FAO) for their energy demand. Apparently, it is the FAO which is diminished in chronic kidney diseases as a result there would be an increased accumulation of fatty acid, thus causing dyslipidemia. Some of the ancillary molecules that are involved in the activation of pathways relevant to inflammation and perturbed fatty acid oxidation conceivably include TGF-β and SMAD. The TGF-β, a cytokine that is upstream of SMAD, and the latter binds to the regulatory region of PPARγ co-activator −1α (PGC1α) promoter, as suggested by Kang et al. (45). The PGC1α is a transcription factor that besides being a master regulator of mitochondrial biogenesis, it is also known to induce the expression of carnitine palmitoyltransferase (CPT1), a rate limiting enzyme for β-oxidation of fatty acids. Thus, it seems that TGFβ-SMAD-PGC1α axis is an important route via which the FAO is regulated with conceivably modulating the pathways relevant to the inflammatory processes. This being the case it is likely that TGF-β/Smad3 pathway may impair fatty acid oxidation by decreasing the transcriptional expression of PGC1-α. In addition, it should be noted that there are certain other molecules which are involved in impaired fatty acid oxidation like, liver kinase beta 1 (Lkβ1) and AMP activated protein kinase (AMPK). These would be additional signaling molecules involved in the activation of pathways leading to inflammation. Interestingly, palmitate treated cells, comparable to fatty acid accumulation, showed an increased expression of Smad3, which results in negative regulation of PGC1-α, and thereby resulting in mitochondrial injury (46). In support of this notion, it has been shown that obesity-induced mitochondrial injury is ameliorated considerably in Smad3 deficient mice.

Accumulation of lipids in renal cells has been linked to insulin resistance, and insulin signaling has a prime importance in proximal tubular epithelial cells (12, 47). In a murine model, deletion of insulin receptor (IR) in proximal tubules has elevated hyperglycemia, conceivably because of increased regional renal gluconeogenesis, where glucose could be derived from non-carbohydrate sources, e.g., renal tubular lipids or triglycerides (12, 47). Apparently, perturbed renal glucose absorption is also observed in diabetic state because of the increased activity of glucose transporters. For instance, SGLT2 handles normally ~80% of the filtered glucose and other ~20% is derived from endogenous sources generated via regional gluconeogenesis (48). In diabetic state, ~40% increase in renal gluconeogenesis has been reported (49), while at the same time SGLT2 mRNA expression increases by ~36% in diabetic state to handle the additional load by the proximal tubular epithelia (50). Overall, total glucose production in the kidney increases in T2D by ~150% leading to glycosuria, however, the exact percentage of the total glucose handled by SGLT2 in such states is not known (48). Interestingly the renal tubular cells in diabetic state exert a compensatory increase in TmG (transport maximum glucose reabsorption) and renal threshold to handle the excess glucose (48). Like the adaptive processes operative in the liver, insulin is known to decrease renal gluconeogenesis as well (12). Overall it seems that in obesity induced T2DM proximal tubules as well-contribute to hyperglycemia *via* accentuated gluconeogenesis, and this notion is well-summarized by Meyer et al. (51).

In addition, it may be worth comparing the impact of deposition of saturated vs. unsaturated fatty acid in the progression of CKD. As discussed above, within the kidney, specifically in podocytes, mesangial cells and proximal tubules, and influxed macrophages, ectopic lipid accumulation leads to local inflammation, oxidant stress, mitochondrial dysfunctions and eventual fibrosis. In this regard, the fatty acids, in particular the “saturated ones,” contribute significantly toward the onset and progression of CKD. In diabetic nephropathy (DN), accumulation of ectopic lipid and lipid intermediates, like palmitate, ceramide, saturated NEFA, derived from other sources, can deposit in renal parenchyma. The main components of deposited lipid droplets in the renal parenchyma include diacyglycerols, fatty acyl-CoA, ceramides, and sphingolipids. This accumulation of “saturated fatty acids” or intermediaries, as described above, leads to the activation of protein kinase C (PKC) and via series of downstream events lead to mitochondrial dysfunction in the kidney (52, 53). In addition, lipotoxic effects of palmitic acid have been observed in proximal tubules via down-regulation of stearoyl-CoA desaturase-1 (SCD-1). The latter is a key enzyme involved in the pathogenesis of lipotoxicity-induced damage to the kidney, since these lipids in a saturated state would have prolonged exposure to renal cells (54). While, overexpression of SCD-1 is known to attenuate fatty acid-mediated cell toxicity (55). On the contrary, in states of progressive CKD, there is a substantial decrease in polyunsaturated fatty acids (PUFAs). Supplementation of Omega-3 PUFA is known to exert substantial renal therapeutic effects, since it would diminish renal lipid accumulation via dampening the Sterol regulatory element binding proteins-1 (SREBP-1) mediated downstream signaling related to lipogenesis (56).

## Abnormal Lipid Metabolism Associated Changes in CKD

### Perturbed Fatty Acids and Triacylglyceride Metabolism

Lipids are an important contributor to kidney pathobiology, and ~50% of the lipids in renal cells are phospholipids. Out of the remaining 50% nearly 20% are triglycerides and 10% are free fatty acids. The kidney is a dynamic organ with high a ATP demand. Since the proximal tubules have a low glycolytic potential fatty acid oxidation contributes to two third of the oxygen used by these cells. Most of the fatty acids in plasma are bound to albumin, and it is the free fatty acids (FFA), which are used by proximal tubules as a fuel. The process of transport of FFA is primarily mediated *via* a transport protein, known as fatty acid translocase (CD36) (57–59). In addition, apical brush border of proximal tubules also contributes toward FFA uptake. Eventually, imbalance between fatty acid oxidation and its synthesis or increased uptake *via* CD36 leads to accentuated triacylglyceride accumulation (57).

**Figure 3:**
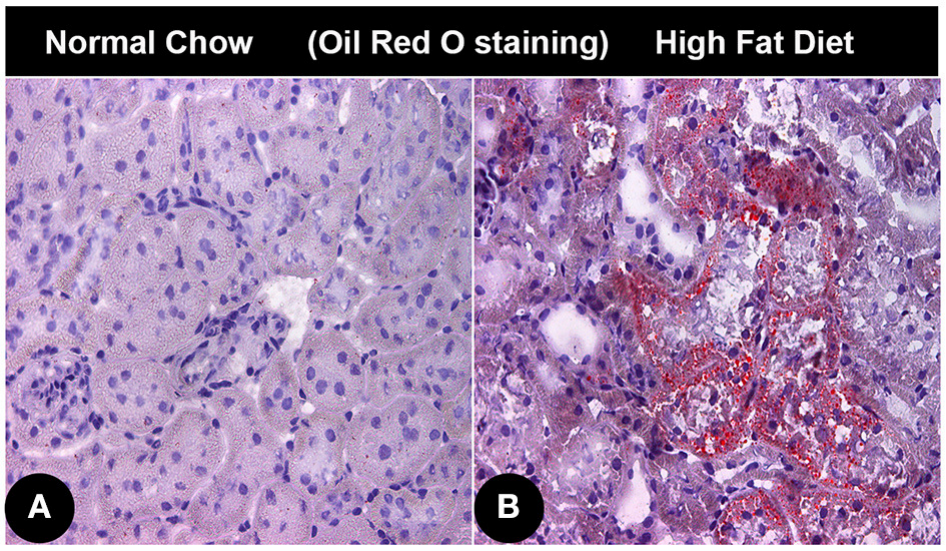
Lipid accumulation in renal cortical tissue of mice with obesity associated diabetic nephropathy. **(A)** Photomicrograph showing Oil red O staining in renal cortical tissue of normal control mice. **(B)** Oil red O stained photomicrograph of renal cortical tissue from an obese mice administered with high fat diet.

SREBPs are the master regulator of fatty acid and triacylglyceride synthesis (58, 60). Mice subjected to high fat diet (HFD) showed an increased expression of SREBP-1, and it is associated with increased accumulation of lipids in renal proximal tubules (Figure 3) (61). Investigations conducted using SREBP-1 transgenic mice suggested that its overexpression leads to increased lipid deposition and chronic kidney disease (CKD). Increased expression of SREBP adversely affect the pathobiology of the mesangium, and influx of inflammatory cells, oxidant stress, and associated increased expression of TGF-β and VEGF. Consequentially there would be an increased expression of extracellular matrix (ECM) proteins leading to glomerulosclerosis, tubulo-interstitial fibrosis with a compromise in renal functions and eventually proteinuria (62). Increased expression of SREBP-1 leads to an accentuated levels of pro-inflammatory cytokines (TNF-α, IL-1β, interferon-γ). The SREBP-1 has also been found to promote the acute inflammatory responses by regulating the genes involved in the production of active IL-1β. In addition, SREBP-1 not only regulates genes involved in lipid metabolism in macrophages, it also activates the expression of core inflammasome NLRP1a via directly binding to its promoter (63). Hence, elevated SREBP expression increases inflammatory responses, and this results in a vicious cycle of fatty acid accumulation, heightened inflammation and oxidative stress. However, besides HFD, hyperglycemia, saturated fatty acid and oxidized lipids, vasoactive peptides like angiotensin II can also increase SREBP-1 levels, resulting in an accentuated TGF-β/Smad signaling and eventually renal fibrosis (58, 64). In support of the above studies are the experiments with SREBP-1 knockout mice, which showed minimal obesity associated renal defects (65).

### Perturbed Cholesterol Metabolism

Imbalance in cholesterol synthesis and its degradation or increased cholesterol uptake and its decreased efflux leads to an accentuated accumulation of cholesterol. Unlike SREBP-1, SREBP-2 is a regulator of cholesterol metabolism (66, 67). Investigations conducted on mice with HFD induced obesity suggest that increased expression of SREBP-2 is intricately associated with cholesterol deposition in renal cells as well as impaired kidney functions (68–70). With the onset of obesity, diabetes or aging, excessive deposition of cholesterol is indicative of increased expression of SREBP-2. Literature data suggest that pro-inflammatory cytokines compromise the functionality of SREBP cleavage-activating protein-SREBP-2-Low density lipoprotein (SCAP-SREBP-2-LDL) receptor, and this leads to perturbations in the 3-hydroxy-3-methylglutaryl-CoA reductase pathway (71). Besides these functional perturbations, it should be noted that oxidant stress in obesity also leads to an increased endoplasmic reticulum stress (ER stress), which results in an enhanced release of SREBP-2 from ER (72). Interestingly, it has been reported that advanced glycation end products (AGEs) via oxidant stress lead to random translocation of ER SCAP to Golgi to undergo glycosylation which may lead to activation of SREBP-2, increased cholesterol synthesis and conceivably impaired renal functions (69).

### Altered High-Density Lipoprotein in Renal Disorders

Under normal circumstances HDL protects organ tissue damage against systemic inflammation *via* removing oxidized lipids from low density lipoprotein (LDL), intermediate density lipoprotein (IDL) and very low density lipoprotein (VLDL). Hyperlipidemia associated nephrotic syndrome is generally characterized by low HDL levels, which at times may be within normal limits (73). However, HDL cholesterol: total cholesterol ratio is reduced in these patients as compared to healthy individuals (74). HDL cholesterol levels are also dramatically reduced in patients with progressive CKD and ESRD. Patient with ESRD have increased levels of oxidized HDL, which exert pro-inflammatory effects, and they bind to large amounts of albumin, phospholipase A2 and serum amyloid A1, and this apparently leads to a compromised HDL functionality (75). One can conceivably deduce that increasing HDL concentration will have potential benefits in ameliorating progression of CKD. Apparently increasing HDL plasma concentration does not reverse advanced CKD. It is actually not the quantity but the quality of HDL particles, which determines its therapeutic potential. It has been noted that HDL particle composition in advanced CKD is abnormal (depletion of large buoyant HDL and enrichment of small dense HDL) (76). In advanced CKD, kidney has fast catabolism of Apo-A1, which means low quality HDL. These low quality HDLs have greater pro-inflammatory effect than the therapeutic ones. Their activity is impaired possibly because of reduced activity of HDL-associated enzymes, such as paraoxonase 1 (PON1), nitric oxide (NO) synthase (NOS) and lecithin-cholesterol acyltransferase (LCAT) (77, 78). In addition, ratio of cholesterol ester-poor HDL3 to cholesterol ester-rich HDL2 is perturbed in patients with advanced CKD (78). The underlying mechanism responsible for the above-mentioned abnormalities in HDL metabolism and structure-functional perturbation are mediated *via* HDL-impaired reverse cholesterol transport system. The impairment in these biological processes also perturbs HDL anti-inflammatory activity (78).

Patients undergoing hemodialysis have marked reduced ability of HDL in removing cholesterol from lipid laced macrophage *via* ATP-binding cassette transporter (ABCA-1), as compared to healthy individuals (78). Irrespective of up-regulation of ABCA-1, rat renal cells display increased accumulation of lipids in 5/6 nephrectomy mode (71). These enigmatic findings rule out the potential role of ABCA-1 in the dysfunctions of HDL structure and reversal of cholesterol transport in advanced form of CKD. However, continuous oxidative damage of ApoA-1 in CKD diminishes the binding affinity of HDL to the ABCA-1 transporter. This leads to an imbalance in HDL mediated cholesterol efflux. These altered cellular processes leads to defective HDL maturation, impaired reverse cholesterol transport and accentuated atherosclerosis in advanced CKD. Malnourished patient suffering from ESRD have hypoalbuminaemia and also have reduced HDL cholesterol levels, which is mainly because of restricted transfer of albumin-bound cholesterol to HDL (79).

Advanced CKD and ESRD not only have HDL deficiency and disrupted reverse cholesterol transport, but also have impaired antioxidant potential of HDL. Patients on hemodialysis also have marked reduction in HDL antioxidant capacity (80). Reduced levels of paraoxnase-1 (PON1) and glutathione peroxidase (GPX) are responsible for reduced anti-inflammatory or –oxidant activity of HDL (78, 80, 81). Impairment in HDL levels in patients undergoing hemodialysis can potentially activate generation of inflammatory cytokines (TNF-α, IL-6, and IL-1β) systemically. Also, increased levels of serum amyloid A1 in patients with ESRD potentiate pro-inflammatory response by impairing the HDL functionality. Incidentally, in healthy controls, HDL is known to cause efflux of endotoxin and serum Amyloid A1 from the blood circulation and minimizes the inflammation since the latter increases the generation and release of pro-inflammatory cytokines (5, 82, 83). That means HDL anti-inflammatory property and the activity of serum amyloid A1 have inverse relationship with each other. Finally, it is well-known that oxidant stress mediated inflammation because of altered functionality of HDL contribute to pathogenesis of kidney disease. Thus, the systemic oxidant stress and inflammation can be regarded as the major drivers of HDL dysfunctions, resulting in subsequent reduction of its anti-inflammatory and antioxidant properties (84, 85).

### Obesity Mediated Inflammation and CKD

Endotoxemia is prevalent in CKD, and this contributes to aggravated systemic inflammation (86). Moreover, it has been suggested that endotoxemia induced alterations in gut microbiome may also contribute to inflammation (87). As alluded above, normally HDL strongly binds to endotoxin and disposes them *via* hepatic route, thus dampening the inflammatory response. HDL deficiency seems to be a prominent factor in aggravating obesity mediated renal changes, as it contributes toward endotoxemia and systemic inflammation that further worsens outcome with microbial infections. Systemic and chronic inflammation are considered as major the factors in triggering CKD in obesity. Mounting amount of data suggest that chronic low-grade inflammation is the dominating feature in this scenario. At times, this could lead to worsening of infections and eventual demise of patients with advanced CKD (88). There are various other factors responsible for chronic low-grade inflammation in CKD, which includes fluid retention and stress induced neuro-hormonal changes leading to an increased activity of the sympathetic nervous system. Subsequently, elevated glucocorticoids and inflammatory cytokines levels would lead to CKD (89). In addition, endotoxemia mediated microbial infection, endothelial activation, oxidative stress, dialysis related comprised immunity, obesity and certain genetic factors also potentially contribute to systemic inflammation-mediated CKD (2, 78). In this regard, it is known that levels of C-reactive protein, pentraxin-3, IL-10 and the ratio of IL-6 to IL-10 increase with the deterioration of kidney functions (90). In addition, chronic inflammation is a major factor responsible for the progression of sarcopenia in CKD. Moreover, acute inflammation in hyperlipidemic state could elevate Ang II levels, resulting in an increased oxidant stress and subsequent endothelial dysfunction, and thus contributing to further renal injury and fibrosis (91). The above-mentioned data substantially affirm the notion that inflammation in obesity contribute toward acceleration of the progression of kidney dysfunctions.

### Altered Renin-Angiotensin-Aldosterone System in Obesity

RAAS is well-known for its role in regulating blood pressure and electrolyte balance (92). Recently, activation of RAAS in local tissues has been the subject of intense investigations (93, 94). Various investigators have suggested the importance of its activation in brain, adrenal gland, heart and kidney. Besides acquiring of angiotensin II (Ang II) in the kidney from circulation, the angiotensinogen (AGT), one of its upstream precursor, has been localized to proximal tubules. Whereas, angiotensin converting enzyme (ACE), that converts Ang I to Ang II, is present abundantly in proximal and distal tubules, and in collecting ducts (93). Renin produced by juxtaglomerular cells in kidney also paves a pathway for localized generation of Ang I. Thus, the kidney has all the necessary elements needed for the conversion of Ang I to Ang II. Ang II binds to type 1 Ang receptor (AT1) in the renal vasculature, leading to vasoconstriction and aldosterone production by the adrenal zona glomerulosa. Excessive release of this hormone is related to oxidative stress and cardiovascular disorders (95). Elevated aldosterone levels are observed in obese individuals, which has a positive correlation with high blood pressure, high waist circumference, and diminished HDL levels (96). Another detrimental factor in obese individuals include that adipose tissue produces inflammatory cytokines like TNF-α and interleukin-6 (IL-6), and these are known to induce insulin resistance as well as to cause aldosterone secretion (93). In addition, conceivably, activation of RAAS system is associated toward accumulation of lipids in adipose tissues (93). In this regard, it is known that AGT is highly expressed in adipose tissues, and with its overexpression these mice were found to be obese (97, 98). Whereas, deletion of AGT protected the mice against HFD-induced obesity (93, 98). These data suggest that inhibitors or blockers of RAAS system can potentially attenuate obesity related hypertension therefore ameliorating renal dysfunctions. Along these lines hypertensive individuals were found to have excessive excretion of urinary angiotensinogen (UAGT), and administration of RAAS inhibitors suppressed UAGT secretion (99). In addition, administration of AT1 blockers lead to a significant reduction in urinary albumin excretion, suggesting a strong link between obesity, RAAS and renal dysfunctions (93).

### Altered Hemodynamic Changes in Kidney

More than four decades ago, patients with obesity associated renal pathophysiological changes were reported (100). Since then, several studies have evaluated renal plasma flow (RPF) in obese and lean individuals. Some of these studies showed 9–33% increase in RPF (12, 101, 102), while others showed no change or rather decrease in RPF (12, 101, 102). In addition, filtration fraction was 9–29% higher in obese participants (101, 103, 104). Interestingly, two of the above studies showed that renal hemodynamic changes appear during the initial stages of obesity when BMI is <30 Kg/m^2^ (12, 102). The degree of increase in RPF was reported to be less than the GFR in obesity, indicating the presence of vasodilation primarily in the afferent arteriole along with vasoconstriction of efferent arteriole. Since it is difficult to calculate the transcapillary hydraulic pressure difference and ultrafiltration coefficient in humans, estimated values for GFR and RPF are the only parameters to roughly measure the changes in these variables (105). Nevertheless, it is known that systemic hypertension is accentuated in obese individuals (104). Conceivably, increased renal vasodilation and transcapillary hydraulic pressure in obese patients contributes to systemic hypertension. Elevated systemic hypertension to a certain extent can lead to hyperfiltration in these obese individuals as the dilated afferent arteriole subjects the glomerulus to an increased arterial pressure; thus accentuating the transcapillary hydraulic pressure difference and eventually elevating GFR in the initial stages of obesity (105). Clinical investigations assessing the changes in renal hemodynamics in patients with severe obesity before and 1 year after bariatric surgery showed a considerable decrease in BMI from 48 Kg/m^2^ to 32 Kg/m^2^ (103) and a considerable improvement in eGFR, RPF, albuminuric state and fractional clearance of albumin. These observations suggest that obesity related nephropathy is a reversible process following weight loss (103).

## Treatment Of Obesity Induced Nephropathy and Possible Therapeutic Targets

Reduction in proteinuria by various interventions yield a reno-protective effect in obesity-induced nephropathy/glomerulopathy. These may include administration of Renin-anigiotensin-aldosterone system (RAAS) inhibitors/antagonists, GLP-1 Receptor agonists, SGLT-2 inhibitors, and weight management either by dietary restriction or bariatric surgery. These are the best studied therapies, besides other possible measures that are still under investigation.

### RAAS Inhibition

The activity of RAAS system can be reduced by several agents, including Angiotensin-Converting Enzyme (ACE) inhibitors, angiotensin type 1 receptor blockers, renin inhibitors, and aldosterone receptors antagonists. Obese patients that were administered ACE inhibitors showed 30–80% reduction in proteinuria (12, 106, 107). In this regard, anti-proteinuric effect of Ramipril, an ACE inhibitor, was more effective in obese and overweight patient with high BMI than those with normal BMI (108). Clinical trials including large series of diabetic patients, showed significant effect of ACE inhibition on delaying the progression to ESRD. These findings suggested that patients subjected to RAAS inhibition for 1 year or longer could prevent CKD progression to ESRD (109). Reports also suggested decrease in the incidence of ESRD with the administration of Ramipril in relatively obese patients as compared to individuals who were mainly overweight. These findings confirm that obese patients are more responsive toward reno-protective effects of RAAS inhibition. Apparently, some of the studies indicated that effects of RAAS blockade tends to wane over a long period, partly because of weight gain in these individuals (108–110), hence these agents have limitated use. Other therapeutic measures may include the use of inhibitors of mineralocorticoids. The mineralocorticoid receptor (MR) activation has deleterious effects on renal structure and functions. In view of this biologic precept, recent efforts were made to establish MR antagonists as a new therapeutic tool. The therapeutic potential of the MR antagonists has been extensively explored in the amelioration of obesity and metabolic syndrome (111). Several preclinical studies suggested the usage of MR antagonist (MRA) have indeed dampened the progression of kidney disease (112). Likewise, a short-term study suggested that supplementation of spironolactone, an MRA, to ACE inhibition therapy significantly reduced albuminuria and blood pressure in obese patients (113). In addition, studies have shown that a third generation non-steroidal mineralocorticoid receptor antagonists like finerenone or eplerenone have potential to abrogate progression of kidney disease and its associated cardiovascular complications (114, 115).

### Weight Loss, *Diet Management*

The effect of diet induced weight loss has been studied in obese patient having microvasculature complications in both non-randomized prospective studies (12) and randomized control trials (RCT) (116). Some of these studies incorporated obese patients with T2DM, and in some trials, patients were subjected to low glycemic index diets accompanied with moderate exercise (116, 117). The BMI of all subjects ranged from 30 to 38 Kg/m^2^ before the trials, and it decreased significantly during the follow up. However, there were notable differences among patients as there were other factors that need to be considered before drawing any concrete conclusions, such as patient compliance and their personal habits pertaining to diet consumption and exercise. Nevertheless, there were remarkable changes related to weight loss and reduction in proteinuria in the above studies, thus establishing a firm causal relationship with one another. Proteinuria levels reduced significantly in patients who had more substantial weight loss. For instance, a RCT showed with weight loss of 4%, there was a 30% decrease in proteinuria, and with further 6–10% reduction in weight the proteinuria decreased by > 70% (12, 118). The meta-analysis of patients with CKD revealed reduction in BMI as well as in proteinuria (119). However, no significant changes in the glomerular filtration rate was observed among all these studies. Besides weight loss, the impact of low glycemic index diet or hypocaloric diets was not only confined to the amelioration of proteinuria, but there was a significant reduction in the elevated blood pressure, hyperlipidemia, fasting blood glucose and insulin resistance (117, 120). In addition, dietary management in obese men with weight loss and achieving waist circumference of <102 cm, also reduced the progression of retino-vascular complications and had improvement in the biomarkers of microvascular endothelial functions (121), suggesting an overall remarkable impact of diet management on obesity related vasulopathies and nephropathy.

### Weight Loss, *Bariatric Surgery*

Mounting amount of evidence indicate a favorable impact of bariatric surgery in obese patients with T2DM (122). Several investigations have suggested a positive effect of bariatric surgery in obesity-associated nephropathy (123, 124). As alluded earlier, influence of bariatric surgery on weight loss or BMI were more dramatic compared to dietary management. An important observation with bariatric surgery in patients with high GFR relates to its significant restoration, and reduction in albuminuria or proteinuria. In addition, these changes were accompanied with considerable improvement in blood pressure, inflammatory and metabolic markers, and these beneficial changes persisted for 1–5 years after surgery (124). Several clinical case reports indicate a considerable amelioration of proteinuria and obesity-induced nephropathy following bariatric surgery (125, 126). However, bariatric surgery was accompanied with other unfavorable renal complications, such as nephrolithiasis, oxalate nephropathy and acute kidney injury (127). Studies have also shown a direct relationship between bariatric surgery and postoperative complications, such as surgical site infections, pneumonia, unplanned re-intubation, and prolonged postoperative ventilation, especially in patients with CKD (128). For instance, the risk of complications was 4.6% in patients with normal renal functions with stage 1 CKD, and the incidence rose to 9.9% in patients with stage 5 CKD. In spite of the beneficial effects of bariatric surgery as indicated above, one cannot conclude that this surgical intervention can reverse CKD or ESRD progression completely (129).

### Glucagon Like Peptide-1 Receptor Agonist

Until now RAAS inhibition has been considered the most effective treatment for obesity related kidney disorders (109). As mentioned before, their therapeutic benefits wane over a long period. Therefore, there is an urgency for finding new therapeutic targets for amelioration of obesity-induced diabetic nephropathy. Newer classes of anti-hyperglycemic drugs have shown encouraging results in obesity mediated renal dysfunctions (129). Recently, GLP-1 agonists showed promising results in dampening the development and progression of kidney disorders and improvement in declining eGFR and reduction in albuminuria (130). These drugs are known to exert beneficial effects in the kidney *via* enhancing glucose induced insulin secretion. The GLP-1 agonists include: Dulaglutide (Trulicity), Exenatide extended release (Bydureon), Exenatide (Byetta), Semaglutide (Ozempic), Semaglutide (Rybelsus), Liraglutide (Victoza), and Lixisenatide (Adlyxin), and within brackets are given the brand names of various manufacturing companies. The degree of weight reduction was different in various GLP-1 receptor agonists. The GLP-1 agonists affect kidney functions in multiple ways besides increasing the insulin sensitivity. For instance, they can induce naturiesis and diuresis *via* inhibiting sodium-hydrogen exchanger (NHE3); conceivably this may indirectly affect the urinary excretion of albumin. Besides modulating albumin excretion, the GLP-1 agonists exert a multitude of other biological effects, for instance increasing the renal plasma flow and dampening the RAAS activation. They are also known to induce marginal improvement in renal hemodynamics. In addition, they have been shown to inhibit production and secretion of intestinal chylomicrons, and hence they have an important functional implication in lipid clearance. Although, GLP-1 agonists play an important role in attenuating the progression of obesity related renal disorders, they are less well-tolerated by patients with ESRD. Certainly, further research is needed to understand the mechanisms by which their effective use can be instituted (130).

Agreeably, anti-hyperglycemic effects of GLP-1 receptor agonist's are-known to reduce the risk of new or worsening kidney disease. Along these lines American Diabetic Association Standards of Care for the treatment of hyperglycemia in type 2 Diabetes state that GLP-1 receptor agonist should be incorporated in the therapeutic regimen if the said metabolic targets are not achieved by metformin in patients with cardiovascular diseases (131). United States Food and Drug Administration recently approve GLP-1 receptor agonist in combinations other drugs, for instance, insulin glargine/exanitide (Soliqua 100/33) and insulin degludec/liraglutide (Xultophy 100/3.6). There were two other trials, i.e., AWARD-7 and LEADER, which have been completed. The AWARD-7 trial pertained to the study of dulaglutide in patients with type 2 diabetes and moderate to severe CKD (131). The LEADER trial dealt with the study of Liraglutide action in patients with diabetes with evaluation and cardiovascular outcome in CKD patients (132). The following table summarizes the list of both the clinical trials in patients with type 2 Diabetes (130, 132, 133):

**Table:** 

**Name of the study**	**Drug or intervention**	**Study population**	**Renal endpoints**	**Renal outcomes**
AWARD-7	Dulaglutide 0.75–1.5 mg vs. insulin glargine	*N* = 577 52% male, Mean age: 65, 52-week treatment	eGFR and UACR change from baseline	Dulaglutide glycemic control was similar to insulin glargine, improved eGFR. Moderate to severe CKD patients showed improved glycemic control with Dulaglutide
LEADER	Liraglutide 0.6–1.8 mg vs. placebo	*N* = 9,340 64% male, Mean age: 64, Median follow-up: 3.84 years	Composite end point New-onset persistent, albuminuria, Persistent doubling of sCr and, eGFR <45 mL/min/1.73 m^2^, Need for continuous RRT, Death due to renal disease	Lower composite renal outcomes were observed with Liraglutide than placebo, primarily reducing the new- onset of persistent albuminuria

*sCr, serum creatinine; eGFR, estimated glomerular filtration rate in mL/min/1.73 m^2^; UACR, urinary albumin-to-creatinine ratio; RRT, Renal replacement therapy*.

### Sodium Glucose Co-transporter-2 (SGLT-2) Inhibitor

Most of the patients with type 2 diabetes mellitus (T2DM) are obese, and that increases their risk of development of cardio-renal complications; thus, sustained reduction in body weight is imperative for a healthy lifestyle (131). Majority of glucose lowering drugs like insulin, sulfonylureas, glinides, thiazolidinediones increase body weight; making weight management difficult in already obese T2DM patients. SGLT-2 blockers are the only glucose lowering drugs, which decrease the body weight. These drugs selectively and reversibly inhibit SGLT-2 transporters in proximal tubules, preventing glucose absorption and increasing its urinary excretion with consequential loss of calories (131). In addition, these blockers decrease insulin:glucagon ratio, thus increasing the degree of lipolysis, leading to weight loss and reduction in fat mass. Recently, a single center Real-World study from India showed mean weight loss of 3.2–3.9 kg with SGLT-2 inhibitors used for 6–12 month (134). In Western population SGLT-2 inhibitor (dapagliflozin) in combination with metformin administered for 24 weeks reduced the waist circumference by −1.7 to −2.7 cm compared to −1.3 cm in placebo (metformin) group (134). Treatment with ipragliflozin for 24 weeks led to a reduction in visceral fat volume and intrahepatic lipid accumulation in Japanese population (131). However, SGLT-2 inhibitors alone are not highly effective in overall body weight reduction, as other counter mechanisms become operative to maintain body weight. Incidentally, SGLT-2 inhibitors in combination with GLP-1 receptor agonists effectively accentuate in the reduction of body weight (131, 135). For instance, dapagliflozin (SGLT2 inhibitor) alone inadequately controlled blood sugar levels in patients with type 2 diabetes undergoing metformin monotherapy (DURATION-8). But when dapagliflozin (SGLT2 inhibitor) was combined with exenatide (GLP1-RA) in patients with T2DM, both effectively reduced the mean body weight (136). Several other investigators have shown that SGLT-2 inhibitors reduce the risk of progression of renal disease in T2D subjects *via* possible mechanisms like reducing glomerular capillary pressure and albuminuria, and therefore attenuating renal impairment (135, 137).

### SREBP Inhibitors

Accumulating evidence have shown that overexpression of SREBP-1 in hyperlipidemic settings is the major driver of tubulo-interstitial injury *via* up-regulating pro-inflammatory cytokines and pro-fibrogenic growth factors (65, 138). These findings led to a search for inhibitors of these cytokines and transcription factors. The farnesoid X-activated receptor (FXR) agonists attenuate SREBP-1 expression, lipid deposition, inflammation and fibrosis in the settings of obesity (130, 139). In addition, vitamin D receptor (VDR) agonists also inhibit SREBP-1, lipid accumulation and thereby reducing fibrosis (67). As mentioned above, SREBP-2 is a regulator of cholesterol synthesis, and exploration of small molecules that can inhibit its transcriptional activity, is the subject matter of ongoing research. Studies have shown that diet management in aged mice and administration of VDR agonists decreased SREBP-2 expression and activity in the kidney (12, 63, 67). This prevented cholesterol deposition and improved declining renal functions (67). In addition, other investigators have demonstrated anti-Inflammatory effects of ANG-(1–7) in ameliorating HFD-induced renal injury via LDLr-SREBP2-SCAP Pathway (140).

### Alternative Approaches

Another strategy, which can limit the accumulation of fatty acids and triacylglycerides, is to increase fatty acid oxidation *via* activation of PPARα. The notion of usage of PPARα agonist is derived from the fact that there is a considerable decrease in PPARα expression and fatty acid oxidation enzymes in kidney biopsy samples of patient suffering from obesity associated nephropathy and diabetes (45). PPARα agonist fenofibrate ameliorated the progression of kidney disease in HFD fed mice (141) and in diabetic *db/db* mice (142). In addition, large-scale studies have shown that elafibranor, a PPARα agonist, reduces the progression of albuminuria, however, there is also transient reduction in GFR. These data suggest that long-term use of these agents may reduce loss of renal functions (143). Another alternative approach to limit the deposition of cholesterol, which can be achieved by accentuating its efflux. Liver X receptor (LXR) has a major role in cholesterol efflux, which conceivably could decrease inflammation and atherosclerosis (144, 145). LXR is known to induce cholesterol efflux in renal cells (146). Renal biopsy samples from diabetic patients with obesity related nephropathy had decreased expression of LXR and of enzymes involved in cholesterol efflux (147). However, the mechanisms behind the accentuated cholesterol deposition, and decreased LXR expression are not fully understood. Inflammation down-regulates LXR expression in renal cells (148). LXR agonist in diabetic mice reduces cholesterol accumulation, inflammation, and finally prevents the progression of renal disease (149). Paradoxically, LXR knockout mice were not protective against diabetic nephropathy (150) as these mice (LXR α/β^−/−^) had increased cholesterol accumulation, inflammation and oxidative stress (150). These reports suggest crucial homeostatic role for LXR in maintaining proper kidney functionality. LXR up-regulation also reduces renin-angiotensin-aldosterone activation (151) and limits ENaC-mediated sodium transport (152), and cystic fibrosis transmembrane conductance regulator (CFTR)-mediated chloride transporter in collecting ducts (153). In line with these biologic precepts, administration of LXR agonist with cyclodextrin has been shown to significantly improve renal function in BTBR *ob/ob* mice (154).

## Conclusion and Summary

Increased BMI not only directly affects renal cells, but also indirectly accentuates kidney damage *via* accelerating hypertension, atherosclerosis and progression of T2DM. Obesity onset increases GFR, RPF and tubular reabsorption of sodium, and also promotes glomerular hypertension. Long standing hyperglycemia accompanied with untreated obesity leads to proteinuria and albuminuria. These changes eventually damage the kidney, and lead to one third of patients developing ESRD. Genetic elements like: gestational diabetes, low nephron number, reduced renal mass, as well as socioeconomic issues have been described as the predisposing factors for obesity associated renal changes, i.e., CKD. Ectopic lipid accumulation in podocytes as a result of uncontrolled obesity also leads to pathologic alterations in renal cells. This leads to progressive albuminuria, oxidative stress and interstitial fibrosis. In addition, lipid accumulation in renal cells leads to insulin resistance. HDL has been regarded as an essential indicator of good health due to its retrograde cholesterol transport, anti-inflammatory and -oxidant properties. Abnormalities in HDL homeostasis leading to hyperlipidemia eventually culminates into the progression of kidney disease, as HDL levels are substantially reduced in majority of kidney disorders. Thus, it seems that interventions improving the HDL status would be one of the good strategies to dampen the progression of CKD. In the regard, one of the other potential therapy would be the blockade of RAAS, although its reno-protective effects are not long lasting. Weight reduction by dietary management or by bariatric surgery has yielded consistent results in limiting the progression of kidney disease. Obesity mediated renal disorder is characterized by altered fatty acid and cholesterol metabolism, and they have important role in modulating inflammation, oxidative stress and fibrosis. Along these lines, the SREBP antagonist, agonist of PPARα, GLP-1RA, SGLT-2 blockers and ongoing clinical trials focusing on dietary intervention hold promise for the treatment of obesity mediated nephropathy. However, we still need more data related to bariatric surgeries to conclude more affirmatively regarding its beneficial effects in CKD. Besides these drug or surgical remedies, we need to explore new therapeutic avenues which could target the abnormalities in fat metabolism to combat obesity related CKD.

**Table:** The table enlists ongoing trials (data collected from ClinicalTrials.gov).

**Intervention/treatment**	**Species**	**Condition**	**Primary kidney outcome**	**Secondary kidney outcome**
Conventional low salt education, Intensive low salt dietary education by smartphone application	Human	Albuminuria	Decrements of 24-h urine albumin levels 12 weeks after low salt diet education	Decrements of 24-h urine sodium, Change of blood pressure
3.6 g of Sodium butyrate, 6 capsules twice daily for 12 weeks.	Human	Diabetes Mellitus, Albuminuria	Change in Intestinal inflammation	Change in urinary albumin-creatinine ratio (UACR), Change in eGFR
Low Sodium Diet	Human	Chronic kidney disease albuminuria	Net change in urinary albumin-to-creatinine ratio	Net change in urinary albumin, eGFR, blood pressure
SGLT2 Inhibition: Dapagliflozine 5–10 mg or Empagliflozin 10 mg or Canagliflozin 100 mg once daily tablet treatment	Human	Diabetic Nephropathy	Change in urinary albuminuria, eGFR, Nephrin, TGF-β1, IL-6, TNF-α	Change in uric acid, aldosterone, renin, angiotensin
Mineralocorticoid receptor Drug: spironolactone 25 mg daily for the 1st or 2nd 6 week treatment period. Drug: Amiloride 5 mg daily for the 1st or 2nd 6 week treatment period.	Human	Chronic kidney disease albuminuria	Change in oxidative stress as measured by urine levels of F2-isoprostanes, albuminuria	Change in serum potassium, glomerular filtration rate
Drug: Sodium zirconium cyclosilicate LOKELMA® 5 gm Powder for Oral Suspension	Human	Type 2 Diabetes Mellitus with kidney complications	Urinary albumin creatinine ratio	Estimation of glomerular filtration rate (eGFR), urinary sodium, potassium
Drug: Sulfa-zero possible benefits of the treatment of new generation hypoglycaemic drugs compared to sulphonylureas	Human	Type 2 diabetes mellitus diet, healthy renal function disorder, albuminuria	Evaluation of glycometabolic parameters, therapeutic adherence, Long term diabetic complications	Evaluation of insulin sensitivity,
Fasting mimicking diet: Prolon Dietary Supplement: Food supplement Endocalyx-4 capsules a day of the food supplement for 3 months.	Human	Diabetic nephropathy, albuminuria, Type 2 diabetes mellitus, glucose metabolism disorders, microalbuminuria	Percentage change from baseline in the microvascular health index to the placebo group	Percentage change in Urine albumin-to-creatinine ratio, urinary heparanase levels, urinary MCP-1 levels, specific urinary heparan sulfate
Drug: Micro-particle Curcumin Three 30 mg capsules once daily, self-administered for 6 months	Human	Chronic Kidney Disease	Percent difference between baseline and 24 week (6 month) albuminuria, Change in eGFR	Renal failure composite, Glycemic control as assessed by change in the percentage of glycated hemoglobin
Behavioral: Coaching DASH group (C-DASH) Behavioral: Self-Shopping DASH group (S-DASH)	Human	Chronic kidney disease, hypertension	Change in Albuminuria	Change in systolic blood pressure

## Author Contributions

IS: writing—original draft, review, and editing. YL, XZ, and YK: review editing. All authors have read and agreed to the submitted version of the manuscript.

## Conflict of Interest

The authors declare that the research was conducted in the absence of any commercial or financial relationships that could be construed as a potential conflict of interest.
